# Genome-wide characterization, phylogenetic and expression analysis of *ABCG* gene subfamily in cucumber (*Cucumis sativus* L.)

**DOI:** 10.3389/fpls.2023.1178710

**Published:** 2023-05-11

**Authors:** Qi Yuan, Jing Zhang, Wanlu Zhang, Jingtao Nie

**Affiliations:** ^1^ College of Horticulture Science, Zhejiang A&F University, Hangzhou, Zhejiang, China; ^2^ Key Laboratory of Quality and Safety Control for Subtropical Fruit and Vegetable, Ministry of Agriculture and Rural Affairs, Hangzhou, Zhejiang, China; ^3^ Collaborative Innovation Center for Efficient and Green Production of Agriculture in Mountainous Areas of Zhejiang Province, College of Horticulture Science, Zhejiang A&F University, Hangzhou, Zhejiang, China

**Keywords:** cucumber, *CsABCG*, bioinformatic character, gene expression, stress responses, microRNA

## Abstract

The *ABCG* is the largest subfamily of the ABC family with extensive functions, and only a few members have been identified in detail. However, more and more studies have shown that the members of this family are very important and are involved in many life processes such as plant development and response to various stresses. Cucumber is an important vegetable crops around the world. The cucumber development is essential for its production and quality. Meanwhile, various stresses have caused serious losses of cucumber. However, the *ABCG* genes were not well characterized and functioned in cucumber. In this study, the cucumber *CsABCG* gene family were identified and characterized, and their evolutionary relationship and functions were analyzed. The cis-acting elements and expression analysis showed that they played important role in development and responding to various biotic and abiotic stresses in cucumber. Phylogenetic analysis, sequence alignment and MEME (Multiple Em for Motif Elicitation) analysis indicated that the functions of *ABCG* proteins in different plants are evolutionarily conserved. Collinear analysis revealed that the *ABCG* gene family was highly conserved during the evolution. In addition, the potential binding sites of the *CsABCG* genes targeted by miRNA were predicted. These results will lay a foundation for further research on the function of the *CsABCG* genes in cucumber.

## Introduction

The ABC (ATP-binding cassette) is a superfamily, one of the largest protein families with transporter activity, most of them are transmembrane proteins and are conserved in organisms (Nguyen et al., 2014). The functional domains of ABC proteins include nucleotide binding domains (NBDs) and transmembrane domains (TMDs). According to the homology of nucleotide binding domains, phylogenetic relationship and topology of the proteins, the ABC family was divided into eight subfamilies, from ABCA to ABCH, of which ABCH subfamily have been shown to exist only in arthropods and zebrafish, but not in mammals, plants and fungi (Annilo et al., 2006; Chen et al., 2011; Dermauw et al., 2013; Dermauw and Van Leeuwen, 2013; Shi et al., 2020). Except for the *ABCG* subfamily, the other seven subfamilies are usually arranged according to the structure of TMD-NBD, while *ABCG* is arranged according to the structure of NBD-TMD (Kos and Ford, 2009). The hydrophilic NBD domain contains highly conserved characteristic motifs, namely WALKER A[GX_4_GK(ST)], ABC signature [(LIVMFY)S(SG)GX_2_(RKA)(LIVMYA)X(LIVFM)(AG)] and WALKER B [(PK)X_2_(RKA)(LIVMYA)X(LIVF)(GA)] (Bairoch, 1992). The ABC signature is the characteristic sequence of ABC transporter, which is an important sign that distinguishes it from other binding proteins. NBD can bind to hydrolyzed ATP to provide energy for transmembrane transport. The hydrophobic TMD domain is composed of 4-6 transmembrane α-helices, which form transmembrane channels and can recognize substrate specificity. According to the composition of NBD and TMD, *ABCG* transporters can be divided into two categories, one is half-size molecular transporter with NBD-TMD domain (White-brown complex, WBC), and studies in drosophila and mammals have shown that this type of transporter needs to interact with itself or another half-size molecular transporter polymerizes to form homodimeric or heterodimeric proteins, which together perform transport functions (Ewart et al., 1994; Tarr et al., 2009). The other type is PDR (pleiotropic drug resistance) type, and the structure is displayed in NBD-TMD-NBD-TMD form, which is called full-size transporter and can function alone.

The *ABCG* is the largest subfamily of the *ABC* gene family, which has important biological functions in plant development and regulation of stress response. During development, it can mediate the absorption and efflux of plant hormones to regulate plant development. For example, the loss of *AtABCG14* expression can lead to severe shoot growth retardation (Ko et al., 2014). And the expression of some *CsABCG* genes were responded to different plant growth regulators and the diterpene sclareolide in cucumber roots (Rajsz et al., 2016). In addition, The *ABCG* also plays an important role in the regulation of disease resistance responses, for example, *AtPEN3* (*AtPDR8*/*AtABCG36*) in *Arabidopsis* conferred non-host resistance to barley powdery mildew (*Blumeria graminis f.* sp. *Hordei*, *Bgh*) (Consonni et al., 2006; Stein et al., 2006). Some *ABCG* genes are highly expressed in the aerial parts of plants and in the roots of plant seedlings to defend against host pathogens when the disease invaded (Mao et al., 2017). Under the induction of pathogen infection or disease resistance signal molecule compounds such as methyl jasmonate, salicylic acid, etc., the expression of many PDR-type transporter-encoding genes was significantly up-regulated, such as *Arabidopsis AtPDR12* (*AtABCG40*) (Lee et al., 2005). Plant secondary metabolites with antibacterial effects such as terpenes, alkaloids and phenols, can prevent bacteria from multiplying in the epidermis. Some PDR-type transporters are indirectly involved in plant resistance to disease attack by participating in the secretion of secondary metabolites (Weston et al., 2012). Some *ABCG* gene families have the function of transporting antibiotics, such as *AtWBC19*, which can localize kanamycin from the cytoplasm to the vacuole, thereby reducing the damage of kanamycin to plant cells (Mentewab and Stewart, 2005). Some *ABCG* gene families can mediate the efflux of heavy metal ions in plants. For example, *AtPDR12* and *AtPDR8* can actively efflux heavy metals in cells, reduce the content of heavy metals in plants and improve the resistance of plants to heavy metal ions (Lee et al., 2005; Kim et al., 2007). In addition, *ABCG* genes also have many important roles in herbicide transport and lignin monomer transport (Hart et al., 1993; Alejandro et al., 2012; Xi et al., 2012).

Cucumbers are one of the most important vegetables in the world, and the *ABCG* genes were not well characterized and functioned in cucumber. Therefore, it is very important to explore the role of cucumber *ABCG* genes. In this study, 33 *CsABCG* genes in cucumber were identified and characterized, and the evolution, expression pattern and predicted binding site by miRNA were analyzed. These will lay the foundation for further study on the function of *CsABCG* genes in cucumber.

## Materials and methods

### Identification of *ABCG* family members in cucumber

Firstly, Search for homologous cucumber *ABCG* sequences on the CuGenDB website (http://cucurbitgenomics.org/) by using the *Arabidopsis ABCG* family proteins as the query sequences (Verrier et al., 2008). Secondly, the domains of *CsABCG* were identified using PF00005 of Pfam (http://pfam.xfam.org/), and *Arabidopsis* homologous proteins were scanned for “hmmsearch”. Through NCBI CD search (https://www.ncbi.nlm.nih.gov/Structure/bwrpsb/bwrpsb.cgi), the *CsABCGs* candidates and their conserved domains were confirmed. And the *ABCGs* in other species were searched using NCBI and confirmed using NCBI CD search (Fiumara et al., 2010).

### Analysis of gene characteristics, genomic distribution, and cis-acting elements in promoters

*ABCG* gene information, including CDS and exon number, was obtained from CuGenDB website and checked with FGENESH (http://linux1.softberry.com/berry.phtml?topic=fgenesh&group=programs&subgroup=gfind). The theoretical pI and molecular weight of the identified *CsABCG* proteins were calculated using the ProtParam website (https://web.expasy.org/protparam/). Moreover, we predicted the subcellular localization of *ABCG* proteins by using the WoLF PSORT website (https://wolfpsort.hgc.jp/). The genome annotation information of cucumber was obtained from Ensembl Plants (http://plants.ensembl.org/index.html). According to the gene position, the physical position of the *CsABCG* genes were mapped using TBtools. Furthermore, cis-element analysis was carried out using Plant CARE (http://bioinformatics.psb.ugent.be/webtools/plantcare/html/) using the promoter sequences (2kb upstream of the gene initiation codon) of *ABCG* genes, and then visualized using TBtools.

### Analysis of the conserved motif and the synteny of the *ABCG* family

Motif prediction of the 33 *ABCG* proteins was performed using the MEME Suite website (https://meme-suite.org/meme/). Results are downloaded in MEME.xml format and imported into TBtools for review. The Ensembl Plant website was used to obtain the genome sequence and annotation of maize, Barley, *Arabidopsis* and tomato. The CuGenDB website was used to obtain the genome sequence and annotation of cucumber, melon and watermelon. Collinear relationships among different species were drawn using TBtools software.

### The expression analysis of *CsABCG* genes

The cucumber transcriptome data (PRJNA80169) was found via the Cucurbit Expression Atlas Cucurbit Genomics Database (CuGenDB), and the tissue-specific expression analysis of 33 *CsABCG* genes were performed and visualized as heatmap using TBtools. Meanwhile, heatmaps of *CsABCG* genes expression were drawn for the cucumber transcriptome data of root development (PRJNA271595), fruit development (PRJNA258122), salt stress treatment (PRJNA437579), powdery mildew treatment (PRJNA321023 (Xu et al., 2017) and data from Nie et al. (2021)), downy mildew treatment (PRJNA388584) and low temperature stress treatment (PRJNA438923).

### Spatiotemporal expression analysis

Tissue-specific spatiotemporal expression analysis of cucumber line 9930 and qRT-PCR were carried out. The root, hypocotyl, cotyledon, stem, leaf, male flower, female flower and fruit of cucumber 9930 were obtained. Three biological repetitions were set up in this experiment, and the tissues or organs of 15-20 cucumber plants were collected as samples in each repetition.

### Cucumber materials and the treatment of different stress conditions

Seedlings of cucumber were grown in a pathogen-free incubator (16/8 h day and night alternation, 25°C) prior to treatment. Cucumber 9930 was used for spatiotemporal and SA- and ethylene-induced expression. A pair of NILs, S1003 and NIL(*Pm5.1*), were used for PM-induced expression pattern analysis (Nie et al., 2015a; Nie et al., 2015b). Leaves of 9930 at the two-leaf stage were sprayed with salicylic acid (SA) (2 mmol/L) and ethylene (200 ppmol/L), and were sampled at 0h, 6h, 12h, and 24h after treatments. For PM inoculation, PM spore suspension (1×10^5^ spores/mL) was sprayed evenly onto the leaves and samples were collected at 0h, 12h, 24h and 48h after inoculation. All experiment set three biological replicates and the leaves of 10-15 plants were sampled at different time points in each replicate.

### Total RNA extraction and qRT-PCR analysis

Total RNA was extracted by Triozol method, and the DNA was removed. The reverse-transcribed cDNA was obtained using PrimeScript First-Strand cDNA Synthesis Kit (Takara, Japan). The *CsActin* was referred as the control. For qRT-PCR, SYBR Premix Ex Taq II kit (Takara, Japan) was used and PCR was performed on a StepOne Plus™ real-time PCR machine (ABI, USA). The qRT-PCR parameters were set to 95°C, 10 s, 60°C, 15 s, 72°C, 25 s, 45 cycles, and default instrument settings were used for melting curve analysis. Quantitative expression analysis was performed using the 2^-ΔCt^ calculus method. Significant differences were determined using Student’s *t* test: *P<0.05, **P<0.01. Primers used in this study is listed in **Supplementary Table S14**.

### Prediction of binding sites of the *CsABCG* genes targeted by miRNA

Downloaded miRNA sequences were from psRNATarget (https://www.zhaolab.org/psRNATarget/) (Dai et al., 2018) and from the data of Nie et al. (2021). The selected cDNA library is “*Cucumis sativus* (cucumbers), cds, Cucumber Genome Sequencing Project, version 2”, and other parameters are set to default values. The track predicted *ABCG* gene binding sites were listed in an Excel spreadsheet.

## Results

### Identification and characterization of the *CsABCG* family genes in cucumber

**Figure 1:**
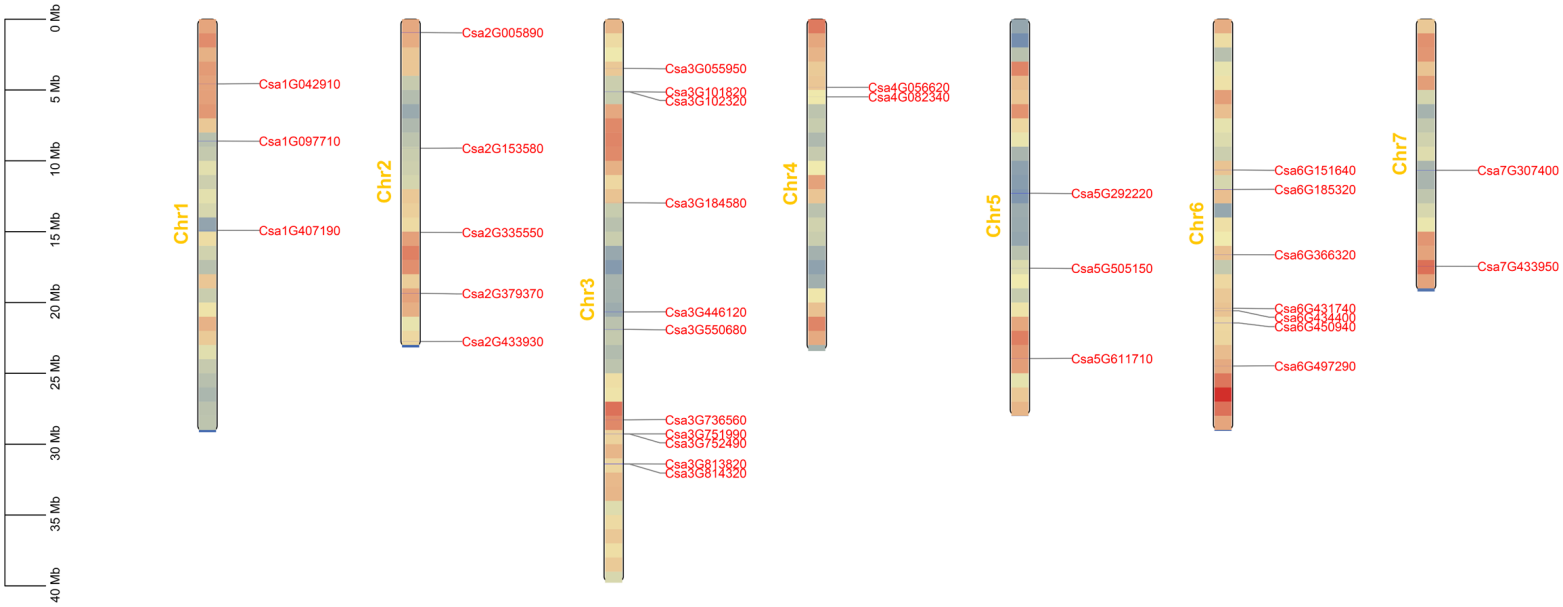
The distribution of the *ABCG* genes located on the chromosomes in cucumber. The genetic distance of seven chromosomes were represented by the scale in megabases (Mb) on the left. The *ABCG* genes are displayed using nomenclature for genome version 2 of Chinese Long cucumber. Gray lines represent the location of the gene on each chromosome.

Using *Arabidopsis ABCG* proteins as the query (Gräfe and Schmitt, 2021), 33 *ABCG* genes were identified in the genome of the cucumber 9930. Their protein sequences were verified using Pfam and NCBI databases for protein functional domains. Based on the modular organization of NBDs and TMDs, The *CsABCG* proteins are classified as half-size transporters (composed of a single copy of NBD and TMD) or full-size transporters (composed of two NBDs and two TMDs). Finally, the *ABCG* gene family was composed of 13 full-size molecular transporters and 20 half-size molecular transporters (**Table 1**). The positions of these 33 *CsABCG* genes were unevenly distributed on the chromosomes of cucumber (**Figure 1**; **Supplemental Table 1**). The majority of the *CsABCG* genes in cucumber were located on chromosome 3 with 11 *CsABCG* genes, followed by chromosome 6 with 7 *CsABCG* genes. Two *CsABCG* genes were located on chromosomes 4 and 7. Among them, 13 full-size *CsABCG* family genes (*PDR*) are mainly distributed on chromosomes 6, 3 and 2, with 4, 4, and 3 genes, respectively. The chromosome 4 and 5 have no *PDR* gene distribution. The half-size *CsABCG* family genes (*WBC*) were mainly distributed on chromosome 3, with 7 genes, and the other *WBC* genes were more evenly distributed on other chromosomes.

**Table 1:** Characteristics of the *ABCG* family of genes and the corresponding proteins in cucumber.

Gen ID	Genomic region	Length of CDS (bp)	Number of Amiao Acicls	Number of Exons	Molecular Weight (KDa)	Theoretical pI	Subcellular Localization	WBC/PDR
Csa1G042910	Chr1 (4569526, 4572775, +)	814	2445	3	91.359	9.18	P	WBC
Csa1G097710	Chr1 (8597235, 8607896, +)	721	2166	11	80.496	9.26	P	WBC
Csa1G407190	Chr1 (1491098, 14917222, -)	1205	3618	20	135.290	6.98	P	PDR
Csa2G005890	Chr2 (936780, 945272, +)	1420	4263	24	161.628	8.64	P	PDR
Csa2G153580	Chr2 (9110042, 9112567, +)	673	2022	5	74.923	9.13	P	WBC
Csa2G335550	Chr2 (15055757, 15057812, +)	617	1854	2	69.527	6.3	P	WBC
Csa2G379370	Chr2 (19355589, 19362608, -)	1404	4215	25	158.914	6.48	P	PDR
Csa2G433930	Chr2 (22750892, 22758878, -)	1451	4356	23	164.516	6.94	P	PDR
Csa3G055950	Chr3 (3474153, 3477341, +)	619	1860	8	69.918	9.13	P	WBC
Csa3G101820	Chr3 (5124258, 5127539, -)	617	1854	4	68.084	9.06	P	WBC
Csa3G102320	Chr3 (5133911, 5138845, -)	609	1830	4	68.096	8.58	P	WBC
Csa3G184580	Chr3 (12957567, 12961277, -)	607	1824	2	67.767	8.46	P	WBC
Csa3G446120	Chr3 (20660805, 20668124, -)	1475	4428	22	166.934	8.88	P	PDR
Csa3G550680	Chr3 (21894045, 21903842, +)	1413	4242	24	160.635	7.58	P	PDR
Csa3G736560	Chr3 (28283076, 28285436, +)	726	2181	2	80.707	9.14	P	WBC
Csa3G751990	Chr3 (29274606, 29277890, -)	672	2019	12	76.059	6	P	WBC
Csa3G752490	Chr3 (29278237, 29282979, -)	552	1659	6	62.819	8.47	P	WBC
Csa3G813820	Chr3 (31382551, 31395010, +)	1428	4287	23	162.224	9.01	P	PDR
Csa3G814320	Chr3 (31400919, 31411219, +)	1451	4356	23	164.696	8.07	P	PDR
Csa4G056620	Chr4 (4811845, 4815798, +)	655	1968	5	72.618	8.65	P	WBC
Csa4G082340	Chr4 (5484261, 5486102, +)	613	1842	1	68.924	9.57	P	WBC
Csa5G292220	Chr5 (12282845, 12296296, -)	735	2208	11	83.872	8.61	P	WBC
Csa5G505150	Chr5 (17578068, 17580720, +)	744	2235	2	82.618	9.41	P	WBC
Csa5G611710	Chr5 (23954696, 23961159, -)	798	2397	11	88.469	5.34	P/C	WBC
Csa6G151640	Chr6 (10646672, 10654617, -)	1443	4332	19	163.751	8.44	P	PDR
Csa6G185320	Chr6 (12008016, 12019660, -)	1430	4293	24	160.195	8.7	P	PDR
Csa6G366320	Chr6 (16617623, 16628441, -)	1484	4455	22	168.187	8.67	P	PDR
Csa6G431740	Chr6 (20410605, 20412622, -)	651	1956	1	73.332	8.98	P	WBC
Csa6G434400	Chr6 (20583000, 20588183, -)	687	2064	9	76.921	9.23	P	WBC
Csa6G450940	Chr6 (21441372, 21449544, -)	1455	4368	20	165.732	8.83	P	PDR
Csa6G497290	Chr6 (24478192, 24480540, +)	714	2145	3	79.948	9.23	P	WBC
Csa7G307400	Chr7 (10660489, 10667772, -)	643	1932	4	70.658	9.16	P	WBC
Csa7G433950	Chr7 (17444035, 17451323, +)	1416	4251	24	161.414	8.84	P	PDR

The “+” and “−” sign indicated the presence of a *CsABCG* gene on the positive and negative strand of that specific marker correspondingly; bp, base pair; P, plasma membrane; C, cytoplasm.

In addition, we predicted the basic physicochemical properties of cucumber *ABCG* family genes and their encoded proteins (**Table 1**). Of all *CsABCG* genes, the CDS is 1659-4455 bp in length and 1-25 exons. The length of *CsABCG* protein is 552-1484 amino acids, and the isoelectric point is 5.34-9.57, and the molecular weights varied significantly, ranging from 62819.37 to 168186.76 Da. In addition, the predicted subcellular localization revealed that except for Csa5G611710, which was located on both cytoplasmic membrane and cytoplasm, the remaining 32 *CsABCG* proteins were all located on the cytoplasmic membrane. It can also be seen that the *CsABCG* genes with closer phylogenetic relationship have the same or similar gene structure (**Supplemental Figure 1**). In addition, the structure of *PDR* genes is more complex than that of *WBC* genes. And the number of amino acids of PDR is generally larger than that of WBC.

### Cis-acting element analysis of the promoters of the *ABCG* genes in cucumber

**Figure 2:**
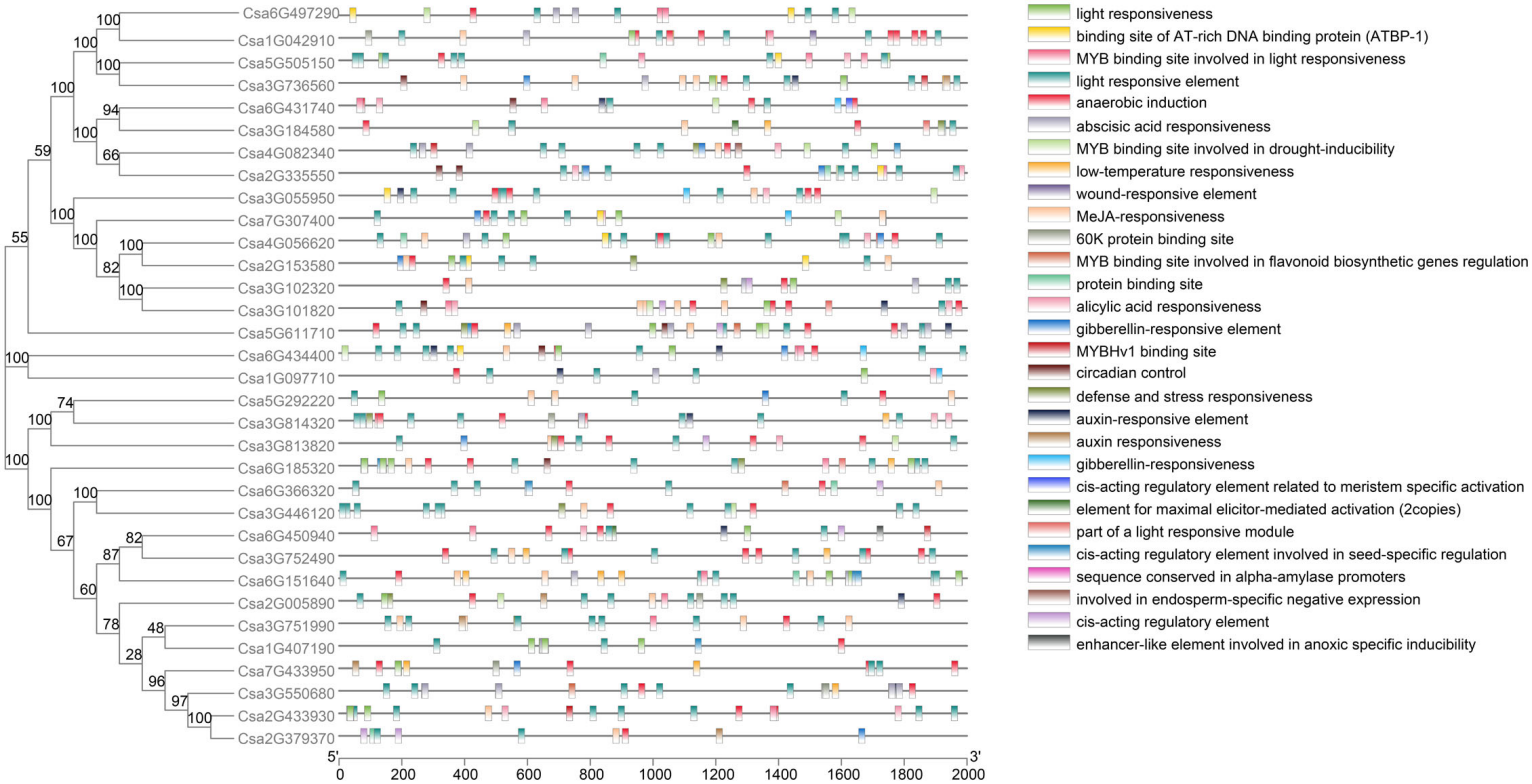
The cis-element of the promoters of the *CsABCG* genes in cucumber.

Cis-acting element analysis was performed on the promoters of the *CsABCG* genes. As a result, more than 20 different cis-acting elements were found (**Figure 2**; **Supplemental Table 2**). The results indicated that the *CsABCG* genes might be involved in the plant development and response to stresses. Many *CsABCG* genes contain hormone-responsive homeopathic elements, of which 16 genes contain gibberellin-responsive elements, 13 genes contain salicylic acid-responsive elements, 23 genes contain abscisic acid-responsive elements, 24 genes contain jasmonic acid-responsive elements, and 5 genes contain auxin-responsive elements. There are multiple cis-acting elements exist in one gene. For example, the promotors of *Csa5G611710* and *Csa2G379370* contains 27 and 9 cis-acting elements, respectively. The number of cis-acting elements contained in different promotors of the *CsABCG* genes is different, which might be related to the function differentiation of the genes. However, genes that contain more cis-acting elements might have relatively complex functions. In addition, the *ABCG* genes also harbor cis-acting elements in response to stresses.

### Analysis of the phylogenetic relationship and the conserved motifs of the CsABCG proteins in cucumber

The gene structures of *CsABCG* genes were first analyzed (**Supplemental Figure 1**). The analysis revealed significant variation in gene structures; for instance, the *Csa6G431740* and *Csa4G082340* genes had only one exon, while the *Csa2G379370* genes had up to 25 exons. Interestingly, *CsABCG* genes in the same clade of the phylogenetic tree exhibited similar gene structures. For example, *Csa6G497290* and *Csa1G042910* had 3 exons and 2 introns, while *Csa6G366320* and *Csa3G446120* had 22 exons and 21 introns.

**Figure 3:**
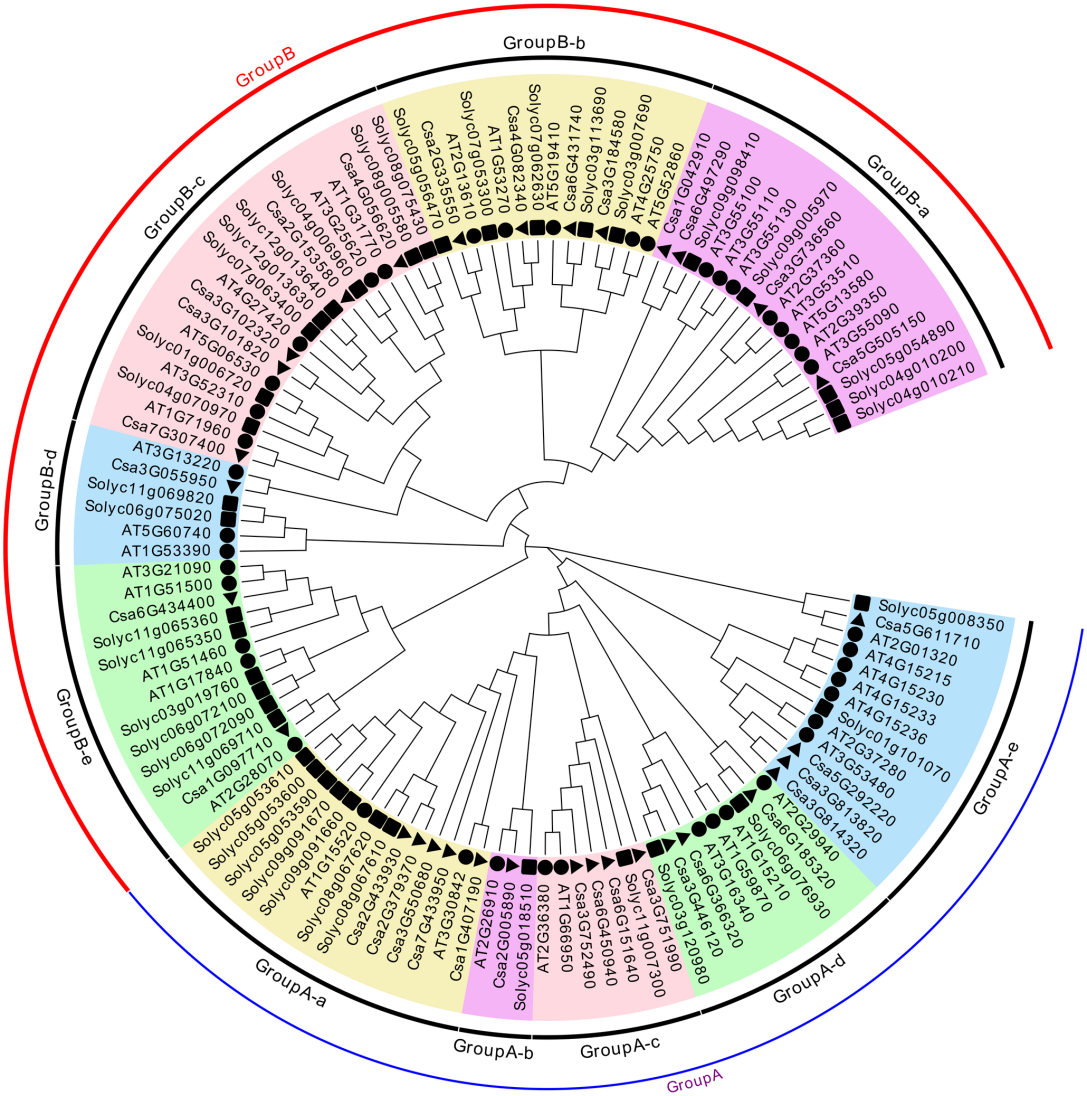
Phylogenetic relationships of *ABCG* proteins among cucumber, tomato and Arabidopsis. The unrooted phylogenetic tree was constructed by MEGA7.0 by neighbor-joining method with 1000 bootstrap replicates. The *ABCGs* are divided into Group A (full-size molecule PDR proteins), and Group B (half-size molecule WBC proteins). The groups A and B can be further subdivided into five subgroups (Group A-a–e, Group B-a–e), as indicated in different colors. The black triangles represented cucumber, black squares represented tomato, and black circles represented *Arabidopsis*.

To further analyze the evolutionary relationship of *CsABCG* family members, a neighbor-joining method was used to construct the phylogenetic tree. The 115 *ABCG* proteins of *Arabidopsis*, tomato and cucumber were mainly divided into two categories, Group A and Group B (**Figure 3**), according to the conserved domains of the *ABCG* family. The Group A is full-size molecule PDR proteins, and Group B is half-size molecule WBC proteins. The groups PDR and WBC can be further subdivided into five subgroups (Group A-a–e, Group B-a–e). The distribution of *CsABCGs* is relatively uniform, and each subgroup has the distribution of *CsABCG* members. For example, GroupA-a has five *CsABCG* proteins. The gene functions and evolutionary relationships among the subgroups were similar, and there was no significant difference in the number of proteins among the subgroups. It can be seen that the *CsABCG* family is relatively conserved during evolution and might be highly similar in function to the orthologous *ABCG* proteins of *Arabidopsis* and tomato (**Figure 3**).

**Figure 4:**
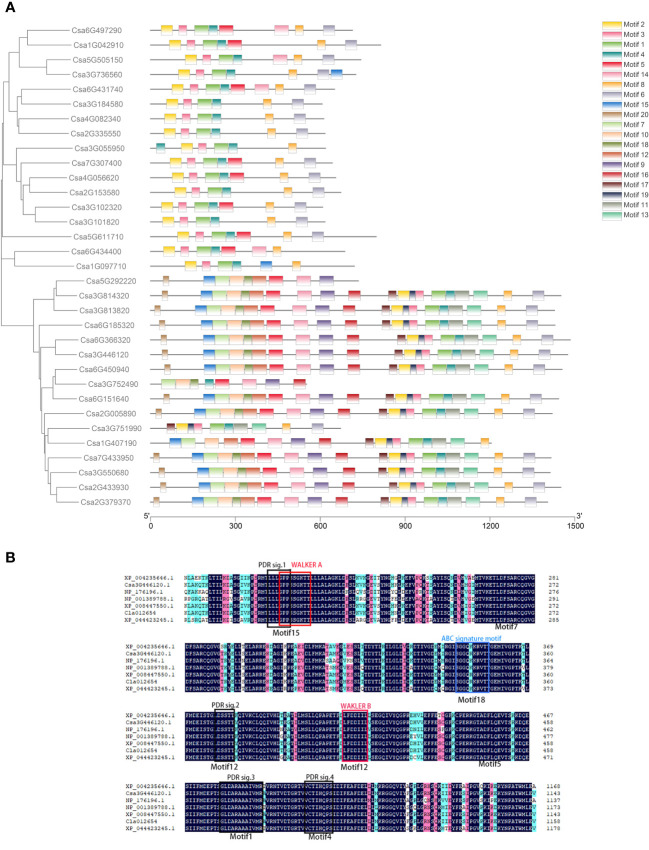
Analysis of the conserved motifs of *ABCG* proteins. **(A)** MEME analysis of the *ABCG* proteins in cucumber. **(B)** Alignment of the conserved PDR domain of partial *ABCG* proteins between cucumber and other species. Black color: the homolog level was 100%; Pink color: the homolog level was greater or equal to 75%; Blue color: the homolog level was greater or equal to 50%. tomato, XP_004235646.1; cucumber, Csa3G446120.1; *Arabidopsis*, NP_176196.1; rice, NP_001389788.1; melon, XP_008447550.1; watermelon, Cla012654; wheat, XP_044423245.1.

According to the amino acid sequences of *CsABCG* proteins, 20 motifs were screened out (**Figure 4**; **Supplemental Figure 2**; **Supplemental Table 3**). It was found that the motifs of *CsABCG* proteins were mainly divided into two categories. The motifs of WBC proteins were mainly arranged in the order of “motif 2-motif 4-motif 1-motif 3-motif 6-motif 7”, and the motifs of PDR proteins were mainly arranged in the order of “motif 20-motif 15-motif 8-motif 10-motif 11-motif 17-motif 5-motif 12-motif 9 -motif 16-motif 18-motif 2-motif 19-motif 4-motif 1-motif 3-motif 14-motif 13-motif 6-motif 7” (**Figure 4A**). Among them, three WBC proteins, Csa5G292220, Csa3G752490 and Csa3G751990, have different evolutionary events, resulting in a protein structure that is more like PDR, and the evolutionary relationship of them was constructed at a position closer to PDR. By aligning the PDR-type protein Csa3G446120 sequence with its orthologous PDR-type protein sequences of tomato, *Arabidopsis*, rice, melon, watermelon and wheat (**Supplemental Figure 3**), it was found that motif15, motif18, motif12, motif1, motif4 are highly conserved in these species (**Figure 4B**; **Supplemental Figure 3**). However, the motifs of some *ABCG* proteins have been mutated or lost during evolution. The 13 PDR proteins contain all 20 motifs, and all 20 WBC proteins have some motifs missing. Alignment analysis of the WBC proteins from seven species showed that the structure of WBC was not as conservative as that of PDR (**Supplemental Figure 3**), which indicated that PDR proteins might play more important roles. There are certain differences in the number of motifs contained in *CsABCG* proteins. For example, Csa4G082340 and Csa2G335550 contain only 6 motifs, while Csa3G814320 and Csa3G813820 harbor all 20 motifs. In addition, proteins that had similar evolutionary relationships harbored more conserved motifs, which indicated that the structure of ABCG proteins is highly conserved during evolution of monocots and dicots.

### Synteny analysis of the *ABCG* genes of cucumber and other species

**Figure 5:**
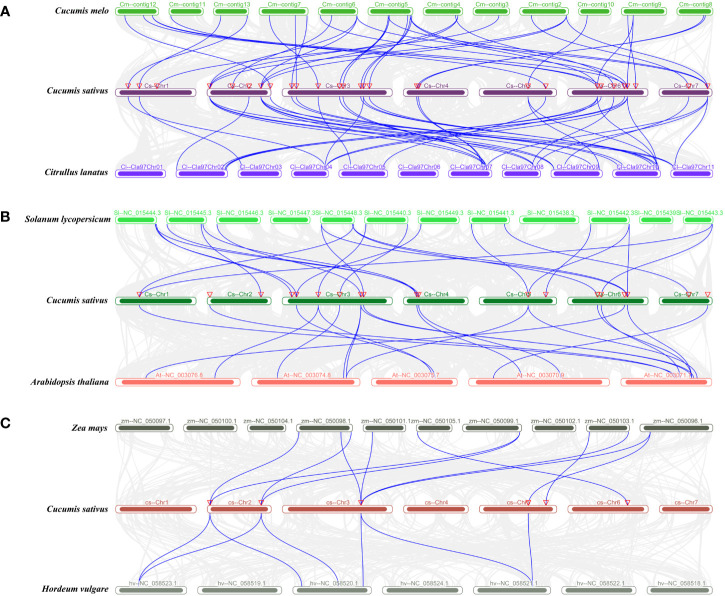
**(A)** Collinearity analysis of *ABCG* genes of cucurbitaceae cucumber, watermelon and melon. **(B)** Collinearity analysis of *ABCG* genes in cucumber and dicotyledons *Arabidopsis* and tomato. **(C)** Collinearity analysis of *ABCG* Genes in cucumber and monocotyledons maize and barley. Synteny of the *ABCG* genes among cucumber and other species. The collinear gene pairs with *ABCG* genes between different species were highlighted by the blue lines. Red inverted triangle indicated the locations of the *ABCG* genes.

In order to reveal the origin and evolution of *CsABCG* family members, we used the MCScanX method (Wang et al., 2012) to analyze the collinearity of *ABCG* genes of cucumber, melon, watermelon, tomato, *Arabidopsis*, barley and maize (**Figure 5**; **Supplemental Table 4**). The results showed that 27 *ABCG* genes in cucumber and 38 *ABCG* genes in melon had collinear relationship. At the same time, there was a collinearity between 26 *ABCG* genes in cucumber and 40 *ABCG* genes in watermelon. Among them, 24 *CsABCG* genes had collinear relationship with the homologous genes of melon and watermelon, which indicated that these 24 *ABCG* genes were relatively conserved in the evolution of cucurbit crops. There was a collinearity relationship between 15 *ABCG* genes in cucumber and 19 *ABCG* genes in tomato. Meanwhile, there was a collinearity between 16 *ABCG* genes in cucumber and 22 *ABCG* genes in *Arabidopsis*. Among them, 10 *CsABCG* genes had collinear relationship with the homologous genes of tomato and *Arabidopsis*, which indicated that these 10 *ABCG* genes were relatively conserved in the evolution of dicots. There was a collinearity relationship between 5 *ABCG* genes in cucumber and 11 *ABCG* genes in barley. Meanwhile, there was a collinearity between 4 *ABCG* genes in cucumber and 7 *ABCG* genes in maize. Among them, 4 *CsABCG* genes had collinear relationship with the homologous genes of barley and maize, which indicated that these 4 *ABCG* genes were relatively conserved in the evolution of monocots and dicots. The above results indicated that the collinearity of *ABCG* genes among cucurbit crops was the most conservative, followed by that among dicots, the least was that among the dicots and monocots. And only two genes, *Csa5G505150* and *Csa3G736560*, had a collinear relationship in cucumber, melon, watermelon, tomato, *Arabidopsis*, barley and maize (**Supplemental Table 4**), indicating that these two genes were the most conserved genes during evolution.

### Expression analysis of the *ABCG* family genes in cucumber

**Figure 6:**
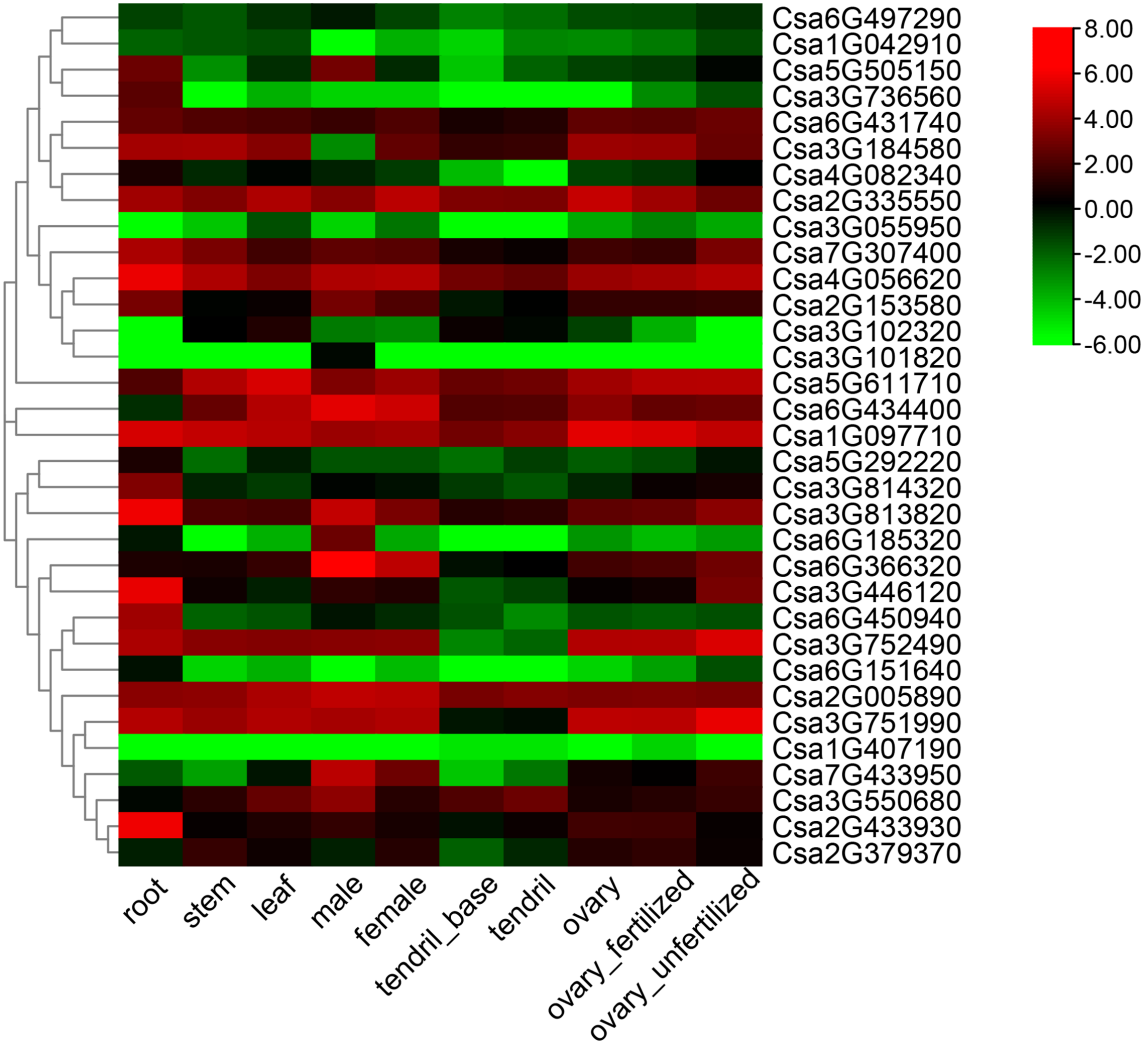
Tissue-specific expression of the *CsABCG* genes in cucumber. The transcriptional levels of *CsABCG* genes in ten tissues or organs of cucumber 9930 were investigated based on public transcriptome data (PRJNA80169). The genome-wide expression of *CsABCG* genes were shown on a heatmap using a log_2_RPKM value, and -6.00 to 8.00 was artificially set with the color scale limits according to the normalized value. The color scale showed increasing expression levels from green to red.

The tissue-specific expression of the 33 *CsABCG* genes was summarized using the RNA-seq data on the CuGenDB website (**Figure 6**; **Supplemental Table 5**) (Li et al., 2011). Some genes are highly expressed in all tissue, such as *Csa2G335550* and *Csa2G005890*, indicating that these *ABCG* genes play important role in the development of cucumber. Some *ABCG* genes were expressed low in all tissues, such as *Csa3G055950* and *Csa1G407190*. Most genes were differentially expressed in tissues, for example the gene expression of *Csa5G505150* and *Csa6G185320* was relatively high in root and male flowers, but was low in other tissues. These indicated that the *CsABCG* genes played different roles in development of cucumber.

**Figure 7:**
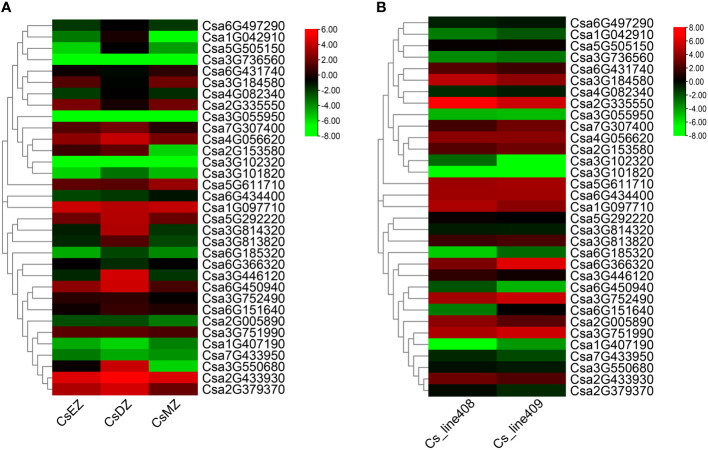
The expression patterns of the *CsABCG* genes in development process of cucumber. **(A)** The expression pattern of the cucumber *CsABCG* genes in root development. CsEZ, root elongation zone, CsDZ, root differentiation zone, CsMZ, root meristem. **(B)** The expression pattern of cucumber *CsABCG* genes in fruit development. The Cs_line408 (long fruit) and Cs_line409 (short fruit) are two near isogenic cucumber lines. The genome-wide expression of *CsABCG* genes were shown on a heatmap using log2RPKM value, and values were artificially set with the color scale limits according to the normalized value. The color scale was shown increasing expression levels from green to red.

According to the cis-element analysis of the promoters of *CsABCGs*, the expression level of *CsABCG* genes might be affected by hormones, biotic and abiotic stresses. To verify whether *CsABCG* genes are involved in the regulation of development and responding to the stresses in cucumber, we summarized the expression model of *CsABCG* genes in previous studies about root development, fruit development, responding to low temperature stress, salt stress, powdery mildew and downy mildew. The transcriptomic data of the differentiation zone, elongation zone and meristem zone of cucumber root four days after planting were visualized (**Figure 7A**; **Supplemental Table 6**). The results showed that *Csa1G097710* and *Csa2G433930* were highly expressed in differentiation zone, elongation zone and meristem of the root. However, some genes such as *Csa5G611710* and *Csa6G431740* were highly expressed only in root meristem. Some genes such as *Csa3G736560* and *Csa3G102320* were expressed at lower levels in the three tissues. These indicated that *ABCG* gene might be closely related to root development. In addition, we found that *ABCG* genes are related to the development of fruit length in cucumber. Comparative analysis of the expression of *CsABCG* genes in fruits of two near-isogenic lines, 408 (long fruit) and 409 (short fruit), were summarized (**Figure 7B**; **Supplemental Table 7**). Most of the *ABCG* genes showed comparable expression level, but some *ABCG* genes are differentially expressed between long and short fruits. For example, the expression levels of *Csa6G366320* and *Csa6G151640* in long fruits are significantly lower than those in short fruits, indicating that *CsABCG* genes might be involved in the regulation of fruit development in cucumber.

**Figure 8:**
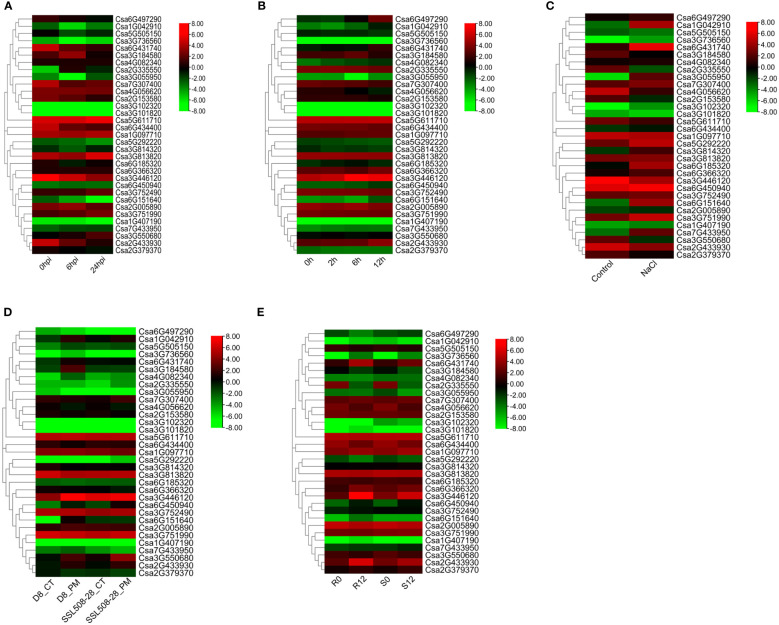
The expression patterns of the *CsABCG* genes under biotic and abiotic stresses in cucumber plants. **(A)** The expression of cucumber *CsABCG* genes in response to DM post-inoculation at 0h, 6h and 24h. **(B, C)** The expression of cucumber *CsABCG* genes in response to chilling stresses and salt stress. Hpi, hours post inoculation. **(D)** The expression of cucumber *CsABCG* genes in response to PM post-inoculation at 0h and 48h. D8_CT, D8 line control; D8_PM, D8 line, 48h post-inoculation with PM; SSL508-28_CT, SSL508- 28 line as control; SSL508-28_PM, SSL508-28 line, 48h post-inoculation with PM. **(E)** The expression of cucumber *CsABCG* genes in response to PM in S1003 and NIL(*PM5.1*). R0, R12: Resistant S1003 at 0 hours and 12 hours after powdery mildew inoculation. S0, S12: Susceptible NIL(*PM5.1*) at 0 and 12 hours after powdery mildew inoculation. The genome-wide expression of *CsABCG* genes were shown on a heatmap using log_2_RPKM value, and -8.00 to 8.00 were artificially set with the color scale limits according to the normalized value. The color scale was shown increasing expression levels from green to red.

Meanwhile, we analyzed the expression patterns of *CsABCG* genes responding to biotic and abiotic stresses (**Figure 8**). Under downy mildew stress, there was no significant difference in the expression levels of the most *CsABCG* genes. But some *CsABCG* genes were up-regulated, such as *Csa2G335550* and *Csa3G550680* (**Figure 8A**; **Supplemental Table 8**), indicating that *CsABCG* genes also functioned in responding to downy mildew invasion. Under low temperature stress, there was no significant difference in the expression levels of the most *ABCG* genes, but some *CsABCG* genes showed an up-regulation trend after treatment, such as *Csa6G497290* and *Csa3G446120*, indicating that these genes play role in regulating low temperature stress (**Figure 8B**; **Supplemental Table 9**). Under salt stress, the expression levels of most *ABCG* genes were up-regulated to varying degrees 12 h after treatment. For example, *Csa1G042910*, *Csa6G431740* and *Csa6G151640* were significantly increased (**Figure 8C**; **Supplemental Table 10**), indicating that these genes might be play an important role in the regulation of salt stress. Some genes had no significant difference at expression level, and these genes might be insensitive to salt stress.

The *CsABCG* genes also responded to PM stress. At the same time point after powdery mildew inoculation (Xu et al., 2017), most *ABCG* genes were not significantly differentially expressed in resistant (SSL508-28) and susceptible (D8) cucumber, a pair of near isogenic lines, but it was worth noting that 48 hours after powdery mildew inoculation, the gene expression levels of some genes in SSL508-28 inbred lines were significantly lower than those in D8 inbred lines, such as *Csa3G184580* and *Csa6G151640* (**Figure 8D**; **Supplemental Table 11**). In addition, another cucumber NIL varieties, resistant materials (S1003, *Csmlo1* genotype) and susceptible materials (NIL(*PM5.1*), *CsMLO1* genotype), showed no significant change in the expression of most *ABCG* genes 12 hours after inoculation with powdery mildew (Nie et al., 2015a; Nie et al., 2015b; Nie et al., 2021). However, the expression of some genes in S1003 was significantly higher than those of the NIL(*PM5.1*) after inoculation with powdery mildew, such as *Csa3G446120* and *Csa2G433930* (**Figure 8E**; **Supplemental Table 12**). Comparing the expression levels of *ABCG* genes after inoculation with powdery mildew of the above four varieties, we found that the expression model of the same *ABCG* gene after powdery mildew infection were also varied in different cucumber combinations. There was no significant difference in the expression of *Csa3G446120* after inoculation between SSL508-28 and D8, but there was significant change in its expression between S1003 and NIL(*PM5.1*). And there was significant change in the expression of *Csa2G433930* at both two groups. These results indicated that the *Csa2G433930* gene played an important role in the powdery mildew resistance of cucumber SSL508-28 and S1003, while the expression of *Csa3G446120* gene might only cause the difference in resistance to powdery mildew between S1003 and NIL(*PM5.1*).

### Analysis of *CsABCG* gene expression by qRT-PCR

#### Tissue-specific expression analysis

**Figure 9:**
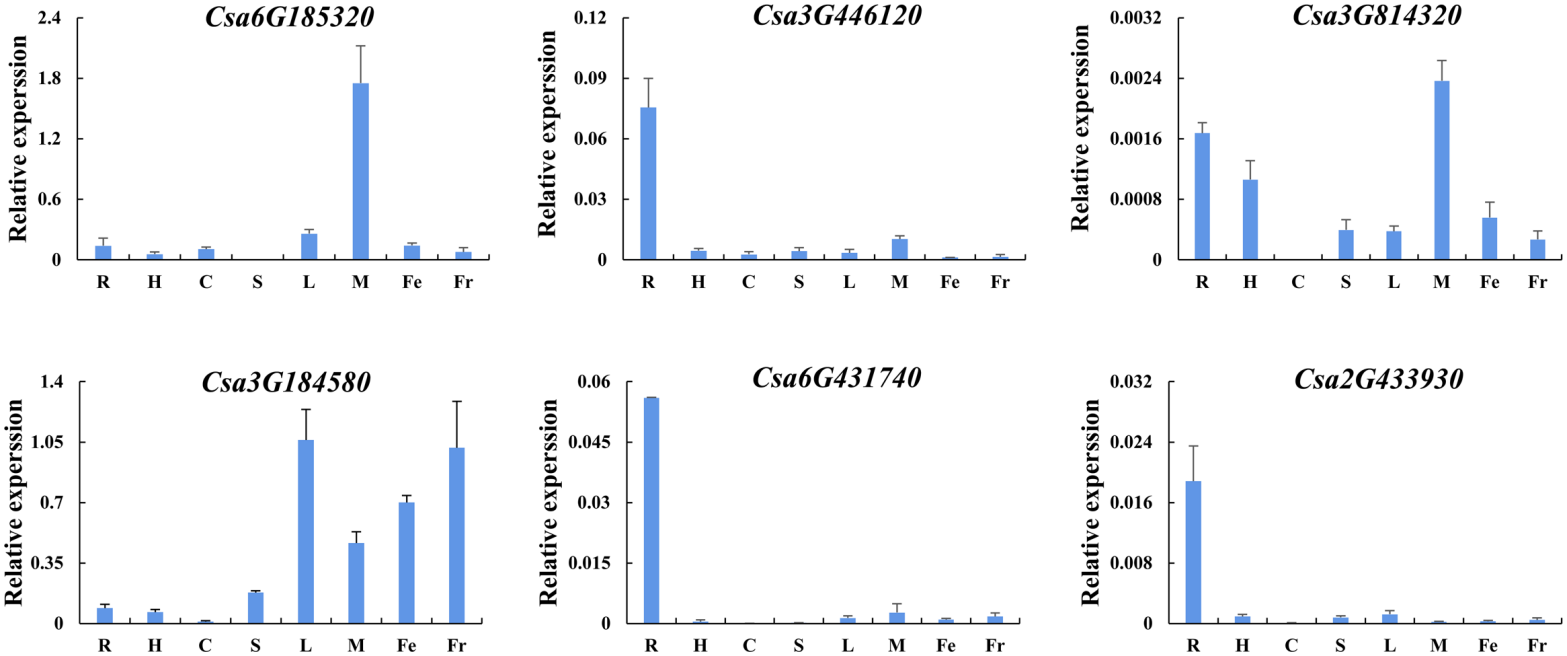
Tissue-specific expression analysis of the *CsABCG* genes in cucumber by qRT-PCR. R, root; H, hypocotyl; S, stem; C, cotyledon; L, leaf; M, male flower; Fe, female flower; and Fr, fruit. The vertical axis is relative to the expression level and x-axis represents different tissues. The *CsActin* gene was used as an internal control for the qRT-PCR. Error bars signify the SD of three biological repeats (n = 3).

In order to further verify the expression patterns of *CsABCG* genes during cucumber development and in response to various environmental conditions, qRT-PCR was performed. Based on the above results, we selected six typical *CsABCG* genes and firstly verified the tissue-specific expression pattern by qRT-PCR (**Figure 9**). The expression of most of these genes was were basically consistent with the above results. The expression of *Csa3G446120*, *Csa6G431740* and *Csa2G433930* were highly in roots, but lower in other tissues. The expression of *Csa3G814320* was highly in roots and male flowers, but relatively low in other tissues. The expression of *Csa3G184580* was higher in leaves, female flowers and fruits, but lowest in cotyledons. The expression of *Csa6G185320* was the highest in male flowers, but lower in other tissues. These results suggest that *CsABCG* gene might play different roles in cucumber development.

#### Expression analysis in responding to PM stresses

**Figure 10:**
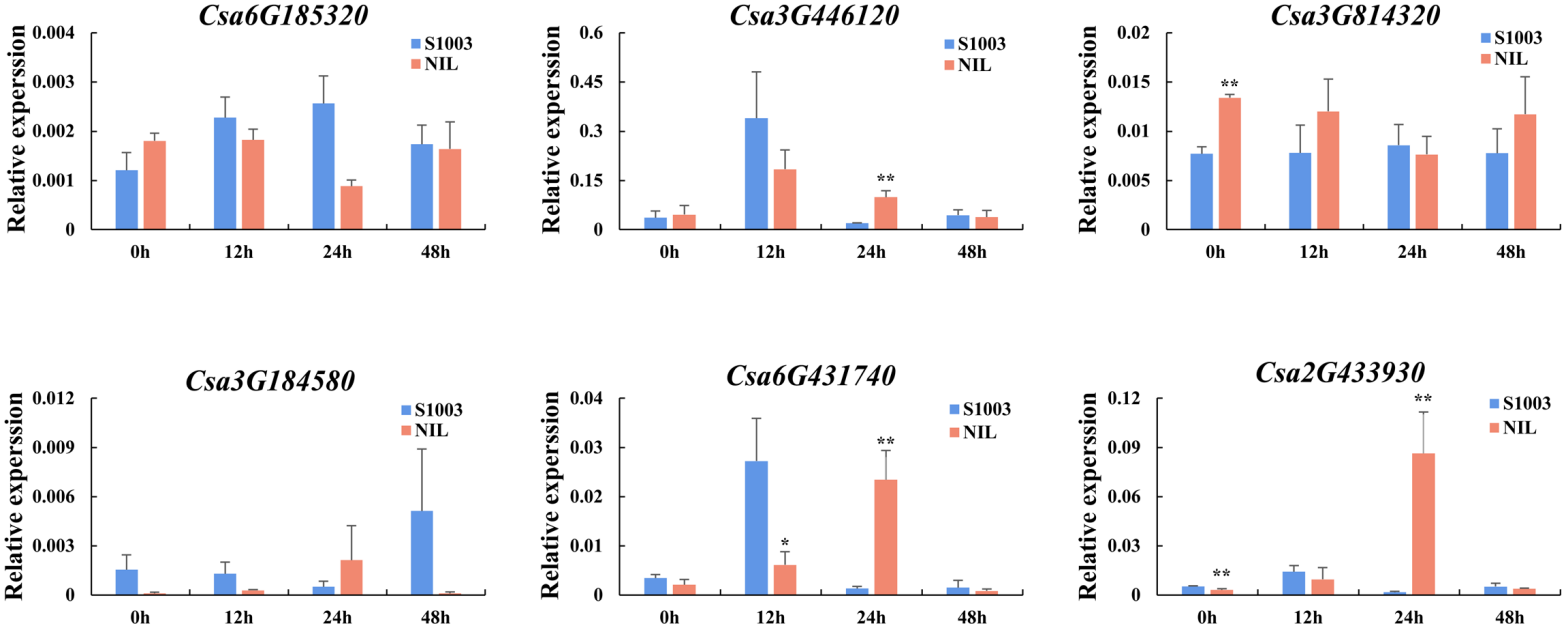
The expression patterns of the *CsABCG* genes responding to PM in cucumber. The expression levels of the *CsABCG* genes were detected in S1003 and NIL(*Pm5.1*) for 0h, 12h, 24h, and 48h after inoculation. S1003, resistance cucumber inbred line; NIL, and susceptible cucumber inbred line NIL(*Pm5.1*). The *CsActin* gene was used as an internal control for the qRT-PCR. Error bars signify the SD of three biological repeats (n = 3). (∗ and ∗∗ indicate significant differences between S1003 and NIL(*Pm5.1*) at *P*= 0.05 and 0.01, respectively).

In addition, we also used near-isogenic lines S1003 (resistant inbred lines) and NIL(*Pm5.1*) (susceptible inbred lines) to detect the expression pattern induced by PM inoculation of the selected genes (**Figure 10**). After inoculation with PM, it was found that the expression pattern of S1003 in *Csa2G433930*, *Csa6G431740* and *Csa3G446120* was similar, and the induced expression of these genes in S1003 reached the highest at 12 hours (h) post inoculation. The expression pattern of NIL(*Pm5.1*) was similar in *Csa3G184580*, *Csa2G433930* and *Csa6G431740*, and the highest expression was found at 24 h. The expression of *Csa3G814320* and *Csa6G185320* was not induced by PM, and only the expression of *Csa3G814320* at 0 h between S1003 and NIL(*Pm5.1*) was significantly different. Except for *Csa3G814320* and *Csa6G185320*, the other four *CsABCG* genes were significantly induced by PM at 12 or 24 h post inoculation in S1003 or NIL(*Pm5.1*), and the expression was significantly different between S1003 and NIL(*Pm5.1*) at different time point after inoculation. Notably, the expression of *Csa6G431740* and *Csa3G446120* in S1003 and NIL(*Pm5.1*) induced by PM was in the same trend, which they were increased and then decrease to the normal level, and the only difference was that the expression in S1003 of these two genes reach the highest level at 12 h but that in NIL(*Pm5.1*) reach the highest level at 24 h. It showed that the expression of *Csa6G431740* and *Csa3G446120* induced more rapidly by PM in S1003 than that in NIL(*Pm5.1*). These results suggest that *Csa6G431740* and *Csa3G446120* might be involved in the of PM resistance conferred by *Csmlo1* (Nie et al., 2015a; Nie et al., 2015b).

#### Expression analysis in responding to hormones

**Figure 11:**
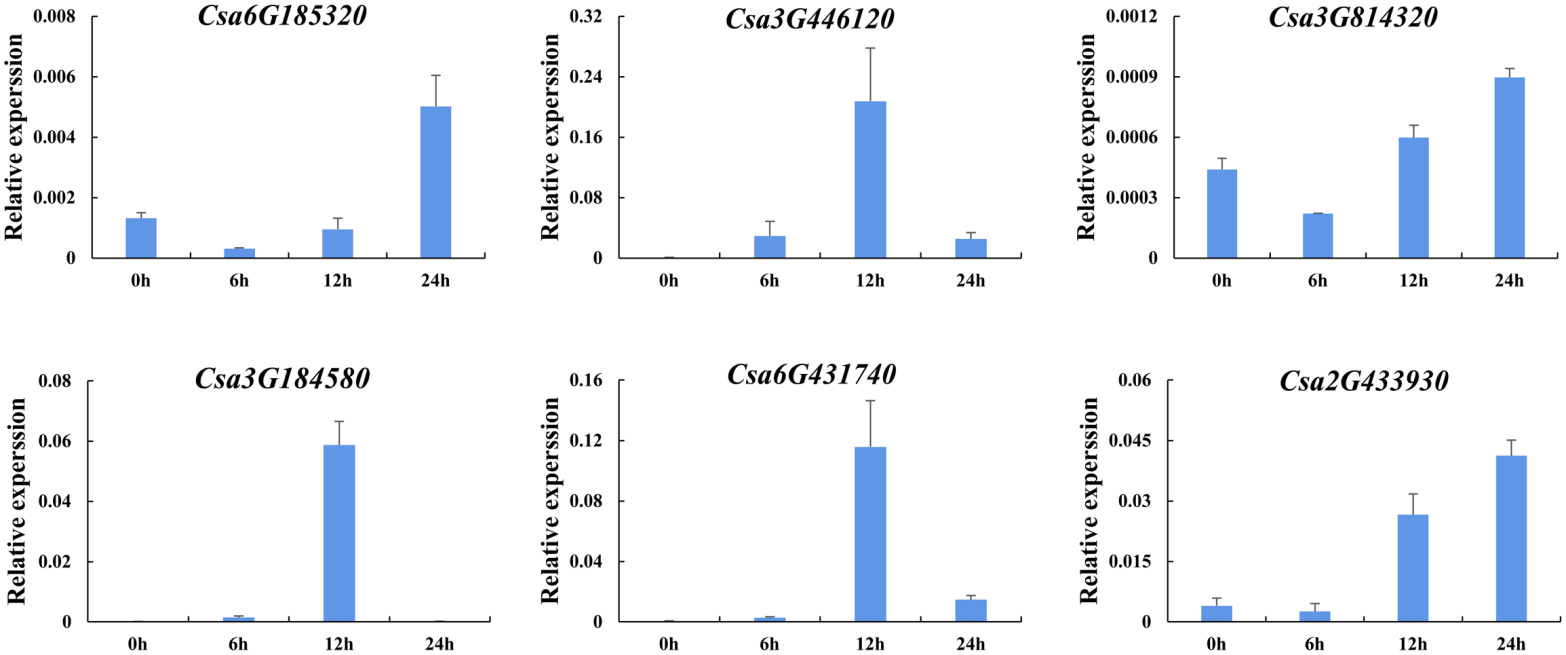
Expression analysis of the *CsABCG* genes responding to ethylene treatment in cucumber. The vertical axis is relative expression level and 0, 6, 12, 24h on the x-axis indicate the treatment time. The *CsActin* gene was used as an internal control for the qRT-PCR. Error bars signify the SD of three biological repeats (n = 3).

**Figure 12:**
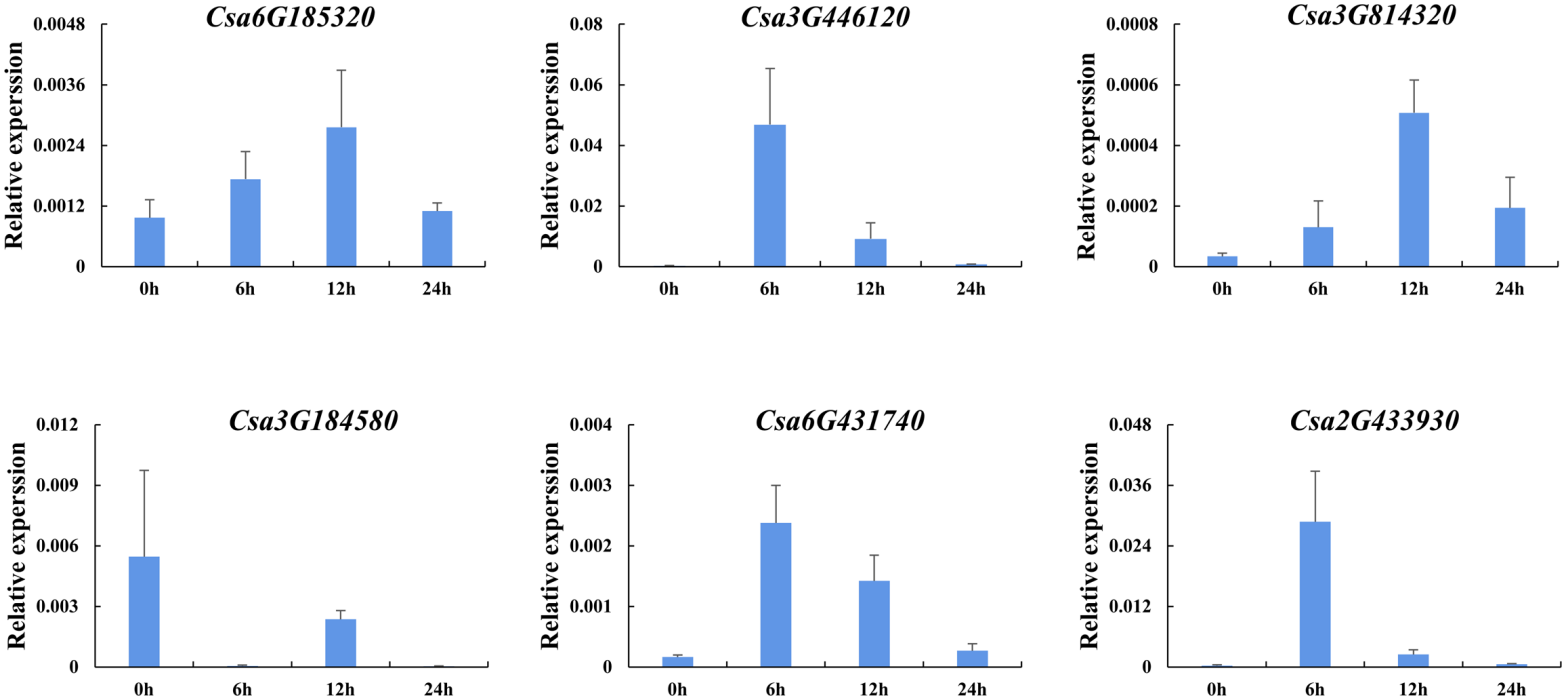
Expression analysis of the *CsABCG* genes responding to salicylic acid treatment in cucumber. The vertical axis is relative expression level and 0, 6, 12, 24h on the x-axis indicate the treatment time. The *CsActin* gene was used as an internal control for the qRT-PCR. Error bars signify the SD of three biological repeats (n = 3).

In order to further analyze the effect of plant hormones that related to stresses on the expression of *CsABCG* genes, the induced expression pattern of the selected genes was analyzed after treated with SA and ethylene using cucumber seedlings. The results showed that the expression level of six *CsABCG* genes changed after ethylene treatment (**Figure 11**). The expression of *Csa6G431740*, *Csa3G446120* and *Csa3G184580* increased at 12 h after treatment and then decreased to normal level. The expression of *Csa6G185320* and *Csa3G814320* decreased at 6 h and then increased, and reached the highest level at 24 h. The expression of *Csa2G433930* increased at 12 h and reached the highest level at 24 h. It is speculated that after exogenous ethylene is applied, the *CsABCG* genes showed different expression patterns, which showed that they played different roles in responding to ethylene. SA also plays important roles in responding to various stresses in different plants. Therefore, SA treatment on cucumber was performed (**Figure 12**). It showed that the expression patterns of the *CsABCG* genes except for *Csa3G184580* had similar responses to SA, all of which increased after treatment of SA and then decreased. The difference was that the expression levels of *Csa6G431740*, *Csa2G433930* and *Csa3G446120* reached the highest at 6 h post treatment, while the expression levels of *Csa3G814320* and *Csa6G185320* were reached the highest at 12 h. These results suggest that the expression of *CsABCG* genes responded to SA treatment and might participant in defending stresses.

#### Prediction analysis of the binding site of the *CsABCG* genes targeted by miRNA in cucumber

miRNAs are a class of eukaryotic endogenous non-coding small RNAs with a length of about 21-24 nt (Park and Shin, 2014), which are widely present in various plants and participate in the regulation of the entire life process such as organ morphogenesis and development (Sun et al., 2022; Zhang L. et al., 2022), hormone secretion (Li H. et al., 2021), signal transduction (Fang et al., 2021), sex differentiation (Zheng et al., 2023) and stress response (He et al., 2021). miRNAs are loaded into the RNA-induced silencing complex (RISC) to modulate post-transcriptional silencing through target RNA cleavage or translational repression (Gregory et al., 2005).

**Figure 13:**
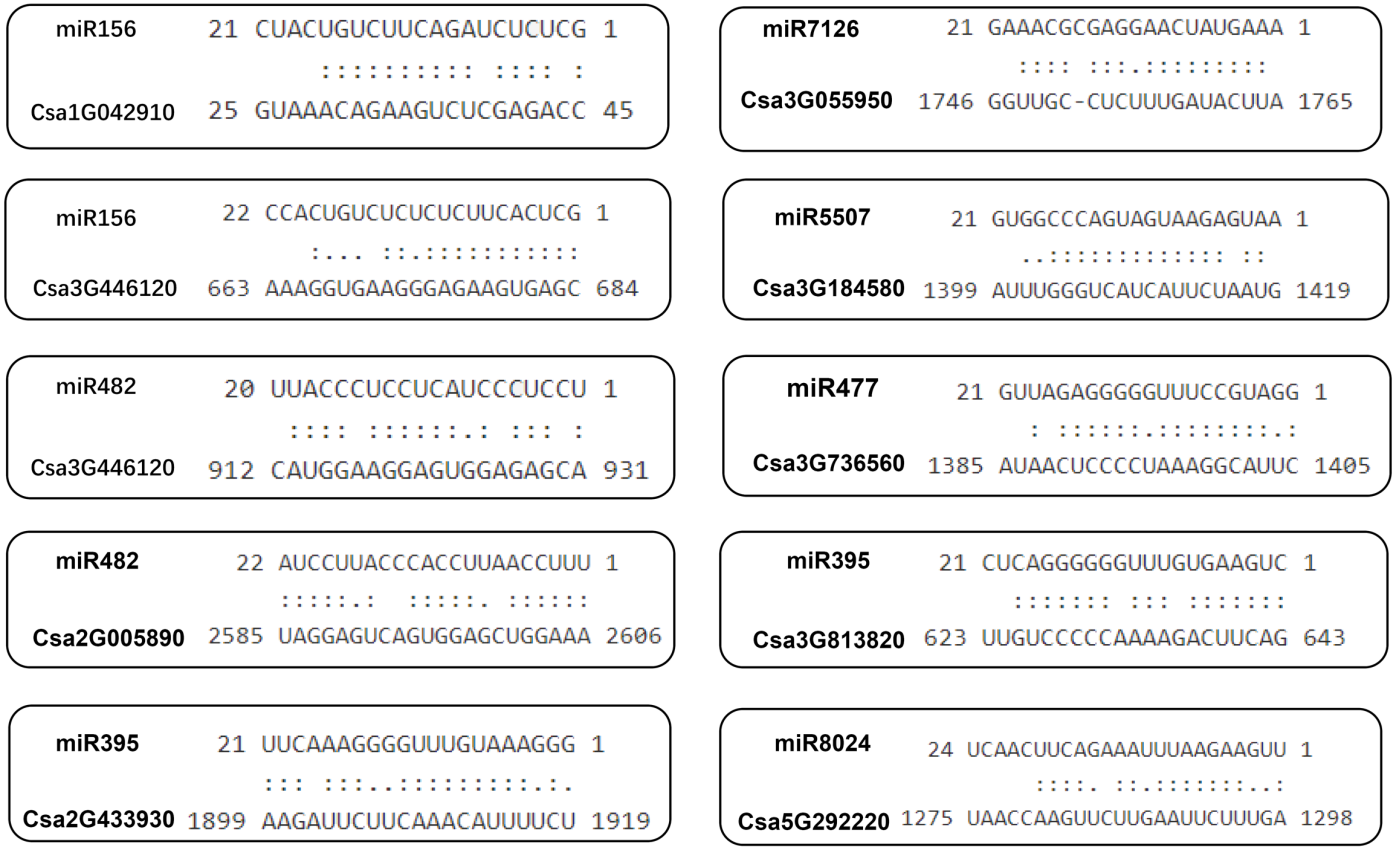
The predicted binding site of the *CsABCG* genes targeted by miRNAs in cucumber. Two dots indicated paired successfully between bases, and one dot indicated that there is also a pairing between U and G in the secondary structure. Blank space indicated that two bases failed to be paired.

To determine whether the cucumber *ABCG* gene is regulated by microRNAs through targeting binding sites, we used the miRNA sequences published on the psRNATarget website to predict the binding sites of miRNAs targeting the *CsABCG* genes (Nie et al., 2021).The results showed that 27 *CsABCG* genes might be regulated by 38 miRNAs. One miRNA could regulate multiple *CsABCG* genes. For example, miR156 could regulate 14 genes including *Csa3G184580*, *Csa3G813820* and *Csa5G611710*, accounting for 42% of total *ABCG* genes; miRNA 396 could regulate 6 genes including *Csa6G450940* and *Csa7G433950*, accounting for 18% of total *ABCG* genes. In addition, five genes including *Csa2G433930* and *Csa3G813820* were targeted by miR159, and five genes including *Csa1G042910* and *Csa2G433930* were targeted by miR166. Likely, one *CsABCG* gene might be regulated by multiple miRNAs. For example, *Csa3G446120* gene has potential binding sites of miR482, miR156 and other miRNAs; *Csa2G005890* gene has potential binding sites of miR172 and miR162 (**Figure 13**; **Supplemental Table 13**).

## Discussion

In this study, 33 *CsABCG* genes were identified (**Figure 1**; **Table 1**), accounting for 0.135% of the total genes in cucumber. The *ABCG* genes have also been reported in other species. There are 51, 39, 43 and 51 *ABCG* genes in pigeon bean, tomato, *Arabidopsis* and rice, respectively (Kusuhara and Sugiyama, 2007; Gräfe and Schmitt, 2021). According to different domains, the ABCG subfamily could divided into two categories, half-size transporters (called WBC) and full-size transporters (called PDR) (Verrier et al., 2008). The number of *PDR* genes accounted for 39.4% of the total number of *CsABCG* genes, and *WBC* genes accounted for 60.6% of the total number of *ABCG* genes. Typical PDR as a whole molecular transporter has a larger relative molecular mass, longer sequence length and more amino acids than those of WBC. Based on the characteristics, we speculate that the *ABCG* gene might be combined the substrates near the membrane and transported to achieve the function of promoting the transport efficiency of substrate molecules. Evolutionary events such as unequal crossovers, insertions/deletions and gene conversions that occur in *ABCG* genes offered the potential to increase mutation rates and formed denser gene clusters on chromosomes, which have been found in the *NLR* gene family of lettuce, radish and cucumber (Chin et al., 2001; Kuang et al., 2004; Zhang W. et al., 2022). In addition, the distribution of genes on chromosomes is uneven. The chromosome 3 harbor the most of *ABCG* genes, and only the chromosome 3 has 3 gene clusters, each gene cluster contains 2 genes. Therefore, it was speculated that the probability of mutant events in the *ABCG* gene in chromosome 3 is higher than that of other chromosomes in cucumber.

Cis-acting elements play roles in important processes such as development, stress response, hormone release, and signal transduction. By analyzing the cis-acting elements of the promotors of *CsABCG* genes (**Figure 2**; **Supplemental Table 2**), we found that there are many important cis-elements in the promoters. Almost all *CsABCG* genes contain hormone-related homeopathic elements and adversity-related cis-acting elements, indicating that the expression of *CsABCG* genes might be related to plant development and responding to stresses. For example, under salt stress, the expression of *Csa1G042910* was significantly up-regulated. During the development of roots, some *ABCG* genes are highly expressed in the root meristem and the least in the root extension region.

Conserved domain analysis was performed on the *ABCG* proteins and 20 motifs were obtained (**Figure 4**; **Supplemental Table 3**), and the fewest number of motifs in transporters contained only 6 motifs. In addition, the number of motifs in WBC transporters is significantly less than those detected in PDR transporters, which is in line with the fact of that the amino acid length of WBC transporter is much lower than that of PDR transporter. Meanwhile, the conserved domain of WBC is less than that of PDR transporter. Collinearity analysis showed that the *ABCG* genes of cucurbit crops maintained higher collinearity due to the closer evolutionary relationship (**Figure 5**; **Supplemental Table 4**). In addition, there were several homologous pairs between cucumber and monocot barley and maize, indicating that these *ABCG* genes might be conserved in both monocots and dicots.

The tissue-specific expression indicated that the cucumber *ABCG* gene has spatiotemporal expression characteristics. And according to the expression analysis, the *ABCG* genes are involved in regulating the development of cucumber roots and fruit growth, indicating that the *ABCG* gene family is closely related to the development of cucumbers. In addition, *ABCG* genes play important role in regulating plant disease defensing. Some *ABCG* genes were induced to higher level after powdery mildew treatment and downy mildew treatment, indicating that they might play an important role in disease resistance. There are obvious differences in the expression of some genes induced by powdery mildew in different cucumber lines. For example, the expression level of *Csa3G446120* in the resistant cucumber (S1003) was significantly higher than that in the susceptible cucumber NIL(*PM5.1*) after inoculation with powdery mildew. While there was no significant difference on the expression of *Csa3G446120* between SSL508-28 and D8 before and after inoculation, which indicated that *Csa3G446120* is not a key disease resistance gene in resistant SSL508-28 and susceptible D8, but is important for powdery mildew resistance in S1003 and NIL(*PM5.1*). The reason for the above results might be that the difference in resistance between S1003 and NIL(*PM5.1*) was caused by the recessive gene, that is, the loss-of-function of *CsMLO1* (Nie et al., 2015a; Nie et al., 2015b), while the difference between SSL508-28 and D8 was controlled by dominant genes, and its candidate target genes are two tandemly arrayed cysteine ​​rich receptor like protein kinase genes (Xu et al., 2016; Xu et al., 2017). The different differentially expressed genes identified in two sets of transcriptomes might specifically participate in the regulatory pathway of the target disease resistance pathways. The *Csa3G446120* is an orthologous gene of *Arabidopsis AtPEN3*, and the involvement of *PEN3* in *mlo*-mediated powdery mildew resistance has been confirmed in *Arabidopsis*. (Consonni et al., 2006). It was found that *Atmlo*-mediated powdery mildew resistance requires assistance of *PEN3*. The *ABCG* genes were also involved in the coordination of abiotic stress. For example, the expression of some *ABCG* genes was significantly up-regulated after salt treatment and low temperature treatment. Among the *ABCG* genes, *Csa3G446120*, *Csa6G431740*, and *Csa2G433930* have been found to play a role in responding to both biotic and abiotic stresses in cucumber. Specifically, these genes demonstrated significant changes in expression levels in response to biotic stresses such as PM and DM, as well as abiotic stresses such as chilling and salt, as indicated by a heat map analysis. Furthermore, the qRT-PCR analysis revealed significant differences in the expression of *Csa3G446120*, *Csa6G431740*, and *Csa2G433930* in response to both biotic (PM) and abiotic stresses (SA, ETH). These findings suggest that these *ABCG* genes could potentially play a crucial role in the response to various types of stress.

MicroRNAs (miRNAs) are essential non-coding regulators of gene expression in plants and animals. In plants, miRNAs guide their effector protein named ARGONAUTE (AGO) to find target RNAs for gene silencing through target RNA cleavage or translational inhibition (Li and Yu, 2021). They play important roles in various stages of life (Cedillo-Jimenez et al., 2020; Qin et al., 2021). For example, miR156 is necessary for transition from juvenile to adulthood (Wu et al., 2009). It showed that miR156 can also combined with target genes to affect the proliferation of spore cells in early stage of anther development, and overexpression of MsmiR156 in alfalfa could lead to longer roots and more nodules (Aung et al., 2015; Zheng et al., 2019). In present study, we found that many *CsABCG* genes in cucumber have potential binding sites of miR156, which indicates that *ABCG* genes might be regulated by miRNA156 and participate in the regulation of plant development. miR482 plays an important role in the development and stress resistance. Furthermore, miR482 was identified as candidate miRNAs for mediating the resistance to tomato *P. infestans* (de Vries et al., 2018). In our study, it was found that there are multiple potential binding sites of *ABCG* genes targeted by miR482, indicating that miRNA482 might be involved in regulating the expression of *ABCG* genes and playing role in disease resistance in cucumber. Polymorphisms of miRNAs and their target sites affect the regulatory functions of miRNAs. More and more evidences have proved that the gain or loss of miRNA functions is closely related to the entire development of plants. For example, the *ZmABCG26* transcript is targeted by zma-miR164. The miR164-*ZmABCG26* module is required for the maize male reproductive process (Jiang et al., 2021). In cucumber, we also predicted multiple binding sites targeted by miR164, but we did not find the potential binding site of miR164 on the orthologous genes of *ZmABCG26*, *Csa4G056620* and *Csa3G055950*, in cucumber. The sequences of *Csa4G056620*, *Csa3G055950* and *ZmABCG26* were aligned, and it was found that the *Csa4G056620* and *Csa3G055950* had multiple mutation sites at the potential binding sites of miR164 (**Supplemental Figure 4**), and these mutated sites might prevent from binding of miR164 to them. This explained why we could not obtain the binding sites of miR164 on *Csa4G056620* and *Csa3G055950*.

## Conclusion

A total of 33 *CsABCG* genes were identified in cucumber. The distribution of *ABCG* genes on chromosomes is uneven, and some genes exist in the form of gene clusters. The corresponding *ABCG* proteins differ in amino acid, theoretical isoelectric point (pI), molecular weight and subcellular localization. The cis-element and expression analysis showed that the *CsABCG* genes were responsible for cucumber development and responding to biotic and abiotic stresses. Analysis of the phylogenetic tree and motifs revealed that the *ABCG* proteins are evolutionarily conserved. Synteny analysis showed that there are more homologous gene pairs for the *ABCG* gene family among dicots than that between monocots and dicots. Likely, cucumber contains more homologous gene pairs with other Cucurbitaceae crops (melon and watermelon) than that with the dicots tomato and *Arabidopsis*. Finally, we predicted potential miRNA-targeted binding sites of the cucumber *CsABCG* genes, and found that the *CsABCG* genes might be regulated by miRNAs. In conclusion, this study lays a foundation for further exploring the biological function of the *CsABCG* genes in cucumber.

## Data availability statement

The original contributions presented in the study are included in the article/**Supplementary Material**. Further inquiries can be directed to the corresponding author.

## Author contributions

QY, JZ and JN conceived the idea. JZ, QY and WZ wrote the first draft. JN corrected the paper to present form. All authors contributed to the article and approved the submitted version.
