# Supplementary material for: Genome-wide characterization, phylogenetic and expression analysis of ABCG gene subfamily in cucumber (Cucumis sativus L.)
Source: Front Plant Sci. 2023 May 11;14:1178710. doi: 10.3389/fpls.2023.1178710 (PMC10211247; doi:10.3389/fpls.2023.1178710)
Supplement: Supplementary file 1 [file DataSheet_1.docx]

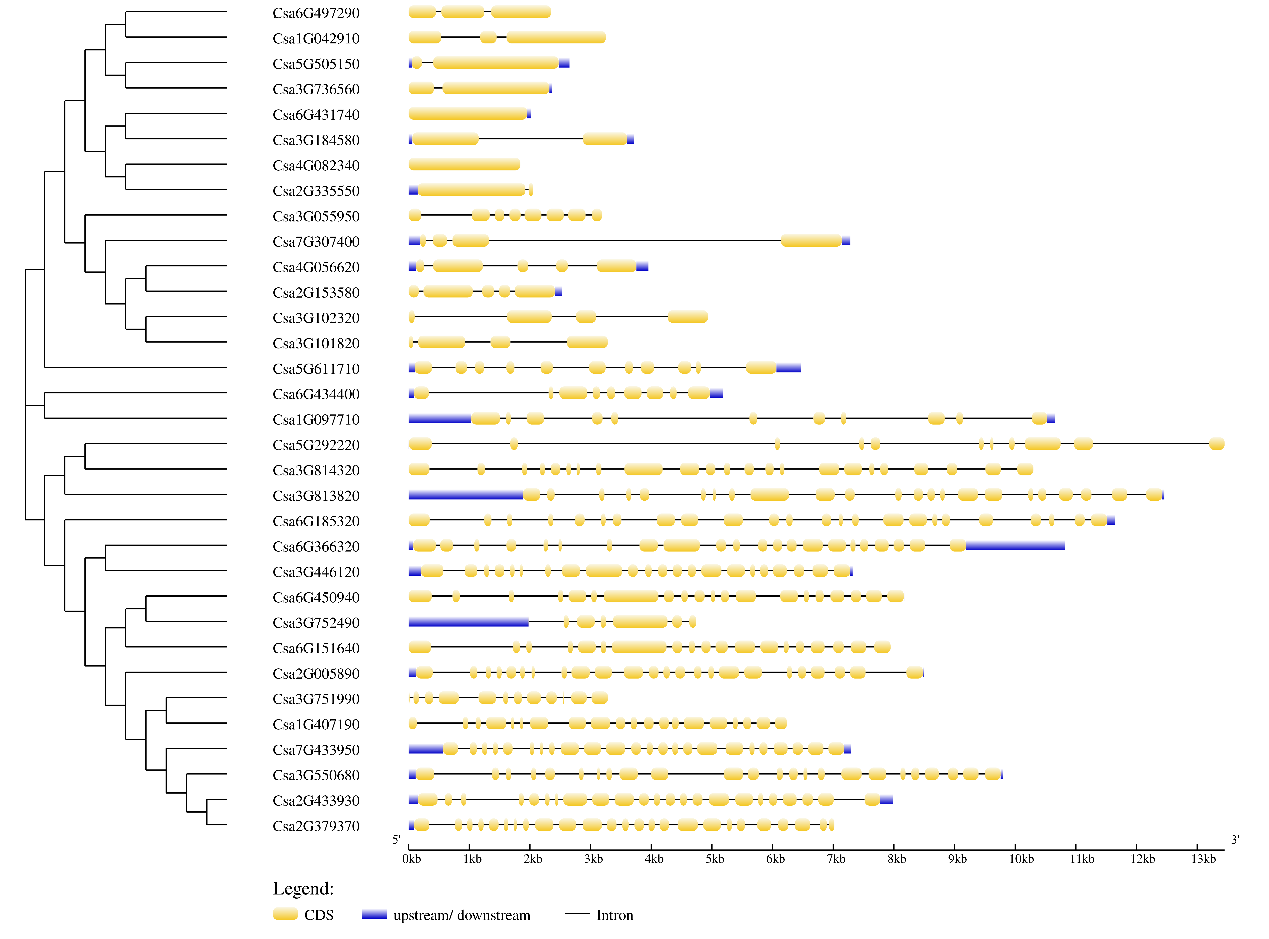


**Supplementary Figure 1 The Phylogenetic tree and gene structure of *CsABCG* genes in cucumber.** Phylogenetic tree was constructed based on the protein sequence of cucumber by MAGE7.0.


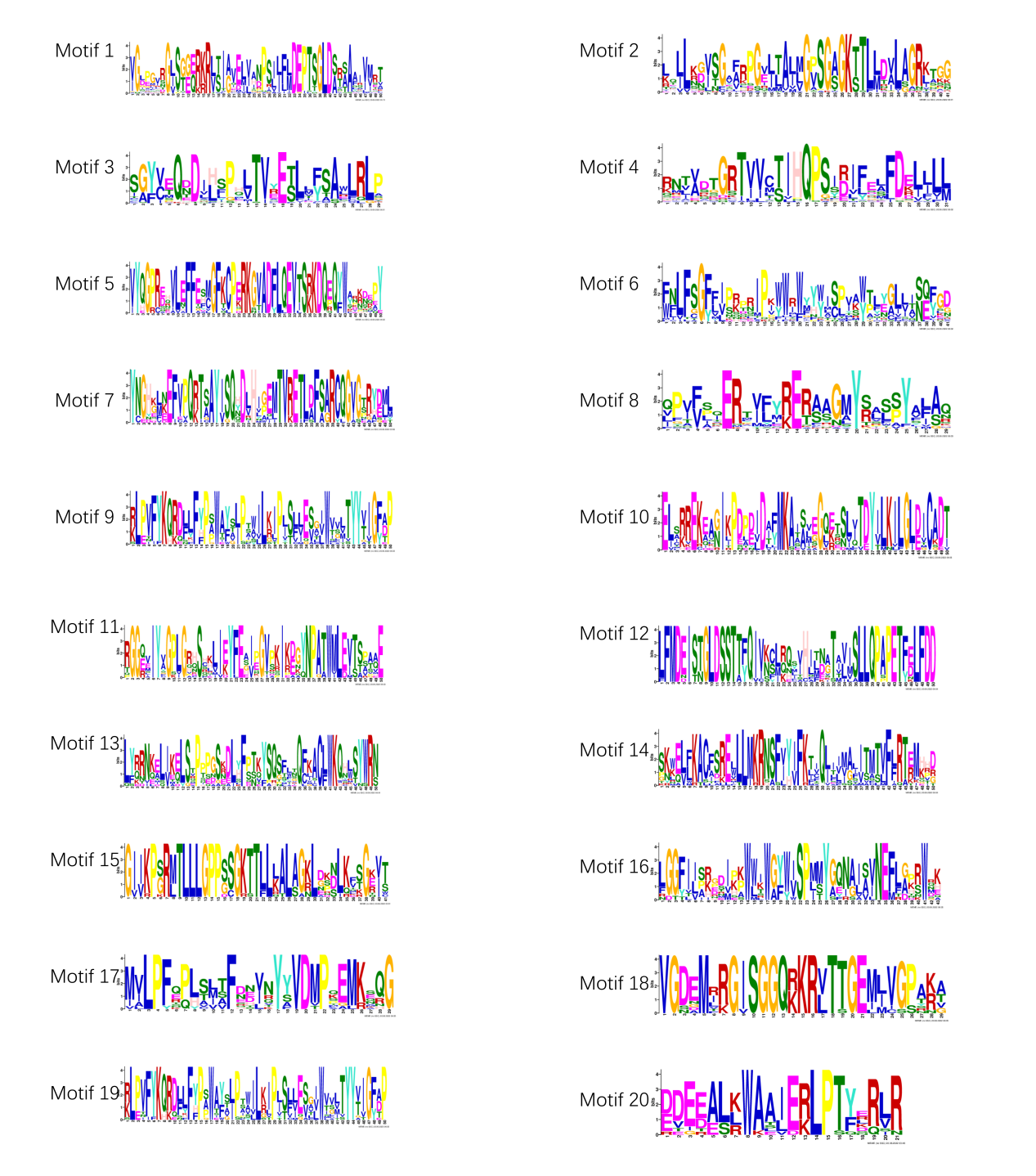


**Supplementary Figure 2 The sequence logos of motifs of the ABCG.**


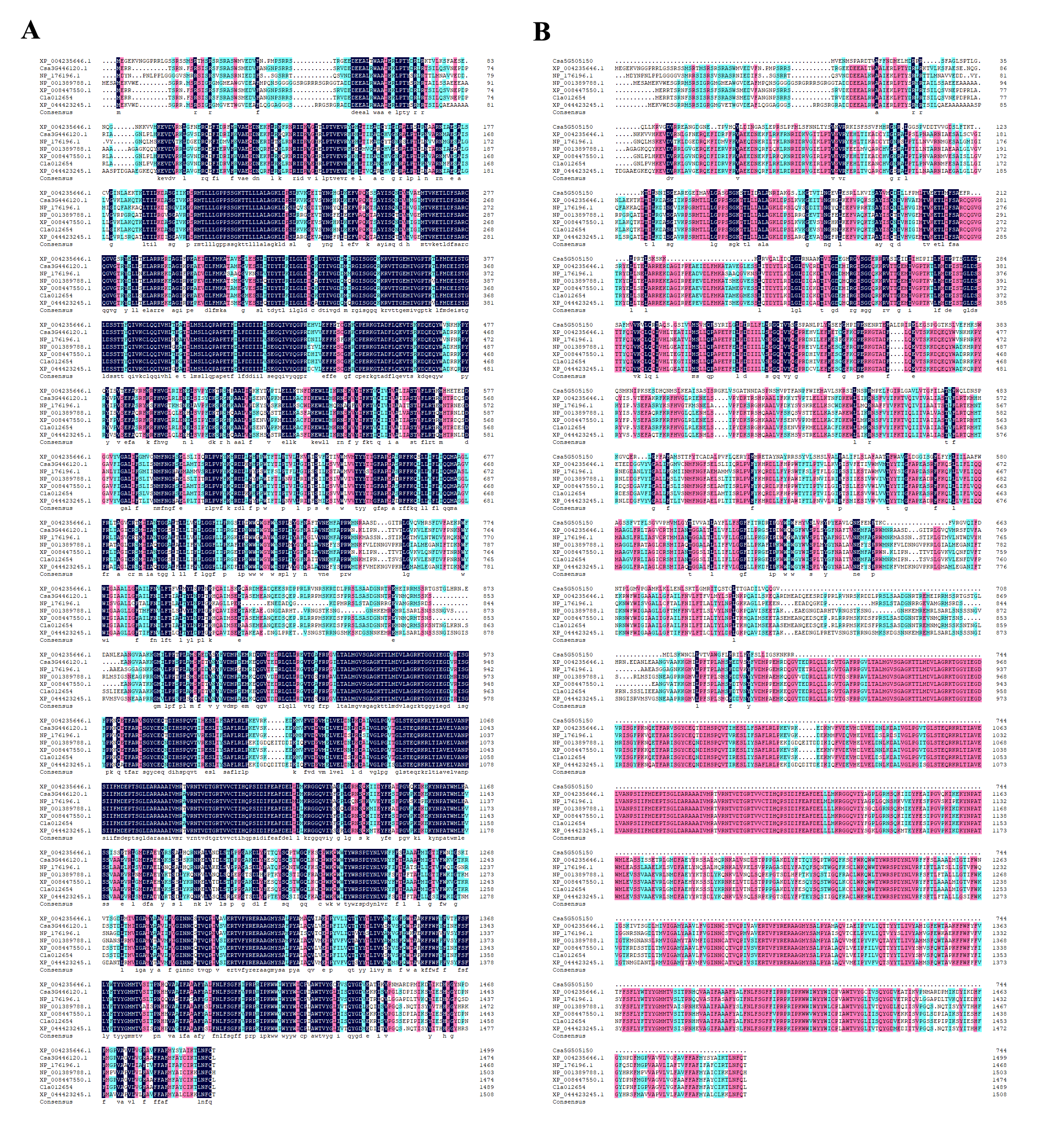


**Supplementary Figure 3 Alignment of the partial ABCG proteins between cucumber and other species. (A) Alignment of the partial full-size ABCG (PDR) proteins between cucumber and other species. (B) Alignment of the partial half-size ABCG (WBC) proteins between cucumber and other species.**


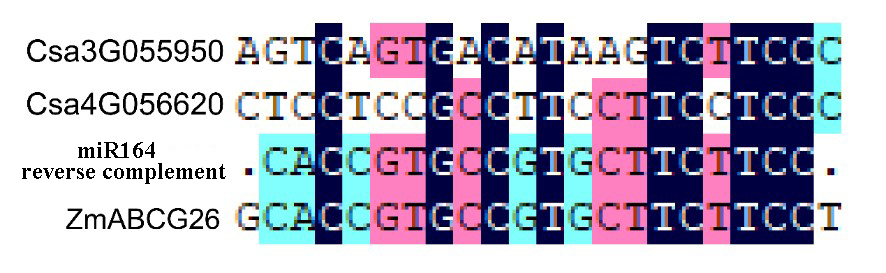


**Supplementary Figure 4 Alignment of the binding sites of the miR164 on *ZmABCG26* and its corresponding sites of the ortholog genes *Csa3G055950* and *Cs4G056620.***
